# Supplementary figures and images for: Neural population dynamics and temporal context cells in macaque medial parietal cortex support temporal order memory
Source: PLoS Biol. 2026 Apr 17;24(4):e3003759. doi: 10.1371/journal.pbio.3003759 (PMC13108878; doi:10.1371/journal.pbio.3003759)

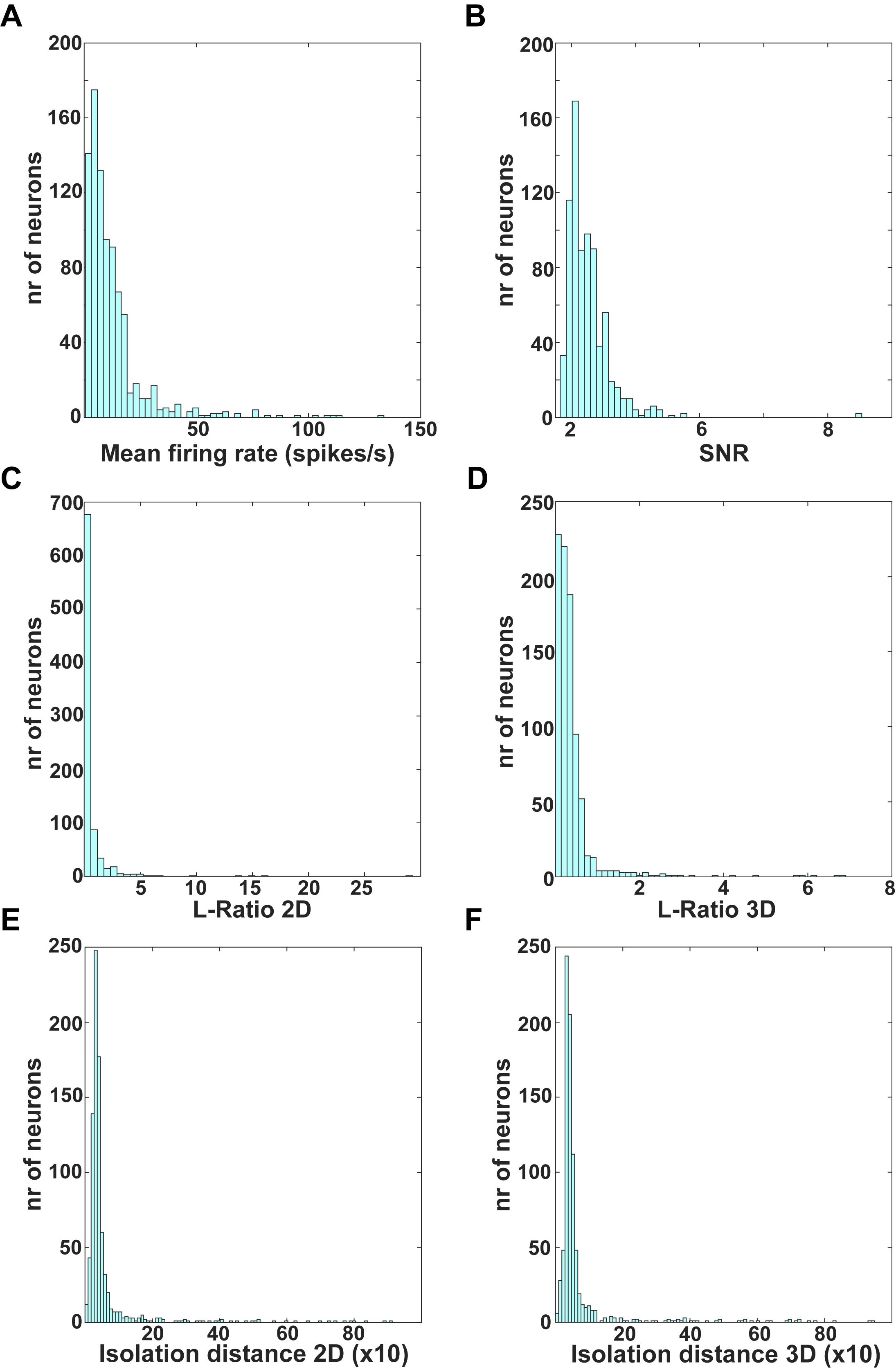

Supplement: S1 Fig — (A) Distribution of mean firing rates for all recorded single units across three monkeys (401 and 275 cells from the main experiment; 198 cells from Experiment 2). The population mean firing rate was 12.29 ± 14.38 spikes/s (mean ± SD). (B) Histogram of signal-to-noise ratios (SNRs) computed from the mean waveform of each neuron (mean ± SD: 2.50 ± 0.73). (C and D) Distributions of L-ratio values calculated from 2D (C) and 3D (D) feature spaces (2D: 0.59 ± 1.56; 3D: 0.39 ± 0.63). (E and F) Distributions of isolation distance computed from 2D (E) and 3D (F) feature spaces (2D: 6.20 ± 10.76; 3D: 7.12 ± 11.49). Together, these metrics indicate that recorded units were well-isolated and met standard criteria for single-neuron quality across experiments. It is associated with main Fig 1. Data underlying this figure is available in S1 Data. (TIF) [file pbio.3003759.s002.tif]

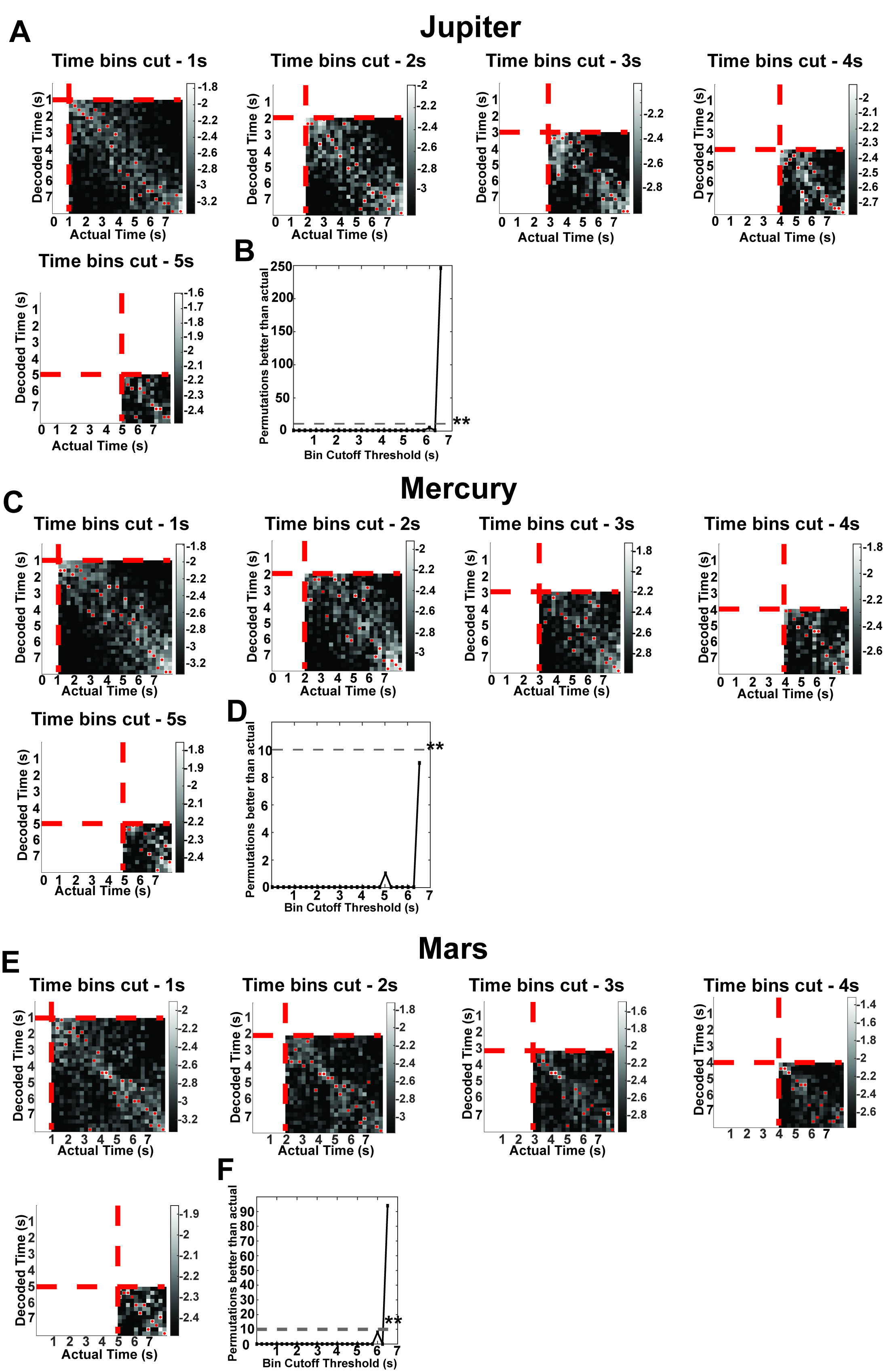

Supplement: S2 Fig — To ensure that temporal decoding was not driven by stronger neural responses early in the encoding period, we repeated the linear discriminant analysis after progressively excluding early time bins from the video in 1-s increments. For each exclusion level, posterior probabilities over decoded time bins and corresponding decoding errors were computed for each monkey. Decoder performance remained significantly better than chance (permutation test, p < 0.01) even after removal of up to 6 s of early encoding activity, indicating that population-level temporal information was distributed across the entire video and not dominated by early visual responses. Posterior probability matrices and corresponding decoding performance are shown for each monkey (A and B: Jupiter, C and D: Mercury, E and F: Mars). For each monkey, the top left panel shows decoding results with the first 1 s removed, with subsequent panels corresponding to removal of the first 2–5 s. Panels B, D, and F show the corresponding decoder errors. The decoded time bin with the highest posterior probability is marked by a red dot, and 1-s intervals are indicated by red arrows. Dashed line marked by ‘**’ denote significance at p < 0.01. It is associated with main Fig 5. Data underlying this figure is available in S1 Data. (TIF) [file pbio.3003759.s003.tif]

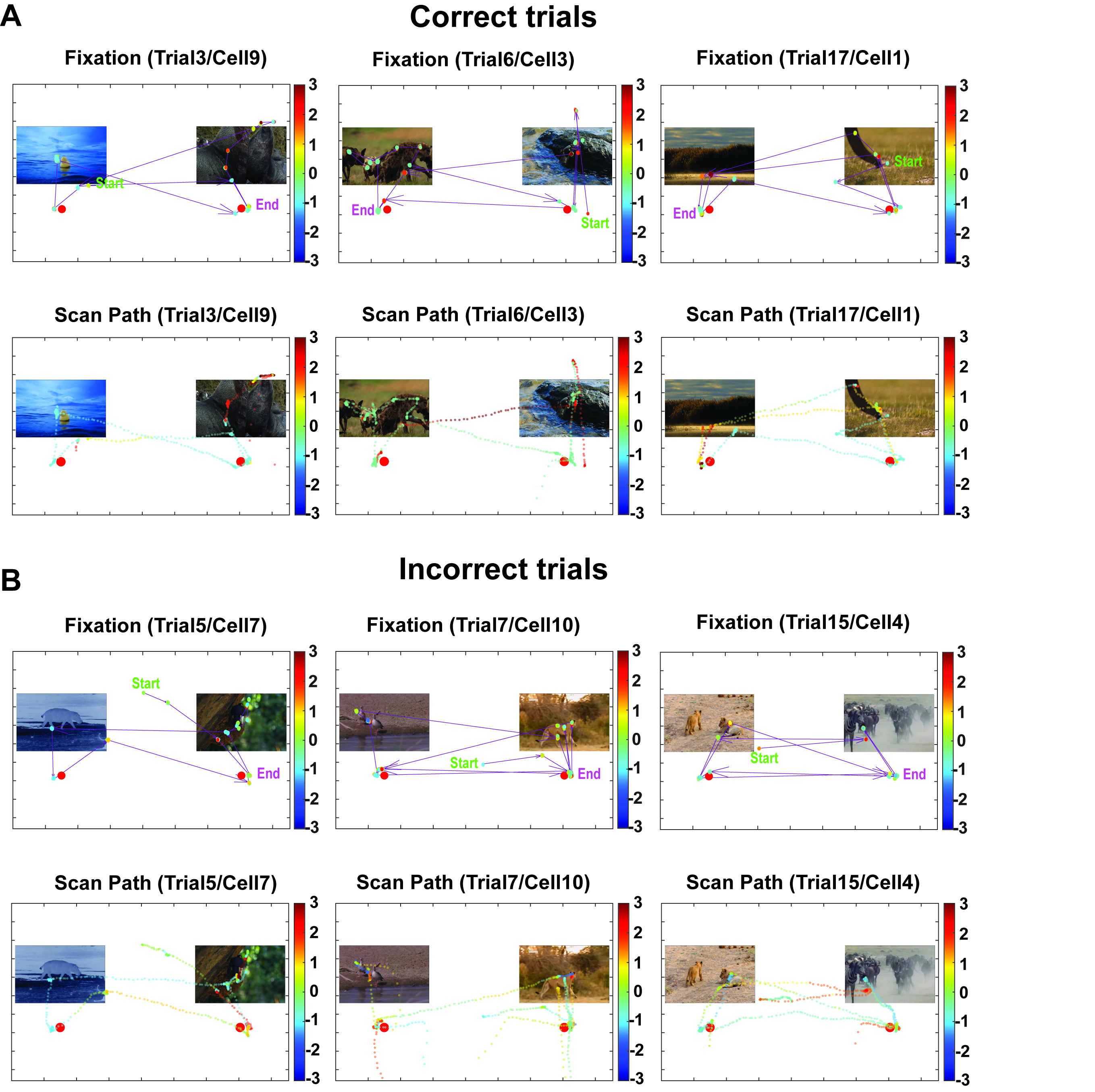

Supplement: S3 Fig — (A and B) Normalized firing rates of six example neurons are shown in relation to eye position during the TOJ period for three correct trials (A) and three incorrect trials (B). For each trial, eye gaze fixations are shown in the upper panels and saccadic scan paths in the lower panels. Neuronal firing rates are overlaid on fixation locations (colored discs) and along scan paths (colored dotted trajectories), with color indicating normalized firing rate (see color bars). The beginning and end of each trial are marked as “Start” and “End”, and arrows indicate saccade direction and amplitude. These examples illustrate that TOJ-related neural activity cannot be trivially explained by fixation location or scan-path, consistent with the GLM-based eye-movement control analyses reported in the main text. All still frames displayed here were generated and assembled by the authors for illustrative purposes and do not contain any third-party copyrighted material. See also S1 Video. Data underlying this figure is available in S1 Data. (TIF) [file pbio.3003759.s004.tif]
