# Supplementary material for: Neural population dynamics and temporal context cells in macaque medial parietal cortex support temporal order memory
Source: PLoS Biol. 2026 Apr 17;24(4):e3003759. doi: 10.1371/journal.pbio.3003759 (PMC13108878; doi:10.1371/journal.pbio.3003759)
Supplement: S3 Table — Summary of experimental models, software, and hardware. The table lists each resource, its source, and relevant identifiers (e.g., model numbers or URLs) to facilitate reproducibility. (DOCX) [file pbio.3003759.s007.docx]

**S3 Table: Key resources used in this study. Summary of experimental models, software, and hardware. The table lists each resource, its source, and relevant identifiers (e.g., model numbers or URLs) to facilitate reproducibility.**

| **REAGENT or RESOURCE** | **SOURCE** | **IDENTIFIER** |
| --- | --- | --- |
| Experimental Models: Organisms/Strains | | |
| Rhesus macaques  (*Macaca mulatta*) | Beijing Institute of Xieerxin Biology Resource | http://www.xexbio.com/cn |
| Software and Algorithms | | |
| SPIKY |  | <http://www.fi.isc.cnr.it/users/thomas.kreuz/sourcecode.html> |
| neuroGLM package for Matlab | Reference^67^ | https://github.com/pillowlab/neuroGLM |
| MATHEMATICA 12.1 |  |  |
| 3D Slicer v 4.10.2 | Reference^68^ | <https://www.slicer.org> |
| Freesurfer |  | <https://surfer.nmr.mgh.harvard.edu/> |
| MATLAB R2020b | MathWorks | https://www.mathworks.com/ |
| Others | | |
| Neural signal acquisition system | NeuroNexus Technologies Inc. | SmartBox system |
| Recording chamber | Gray Matter Research | 32-channel tangential system |
| Eye tracker | SR Research | Infrared EyeLink 1000 Plus |
| Touch sensitive screen (Main experiment) | Shenzhen Anmite Technology | An-190W01CM |
| LiquidCrystal Display monitor (Experiment 2) | LG | 32GP83B |
| Liquid reward dispenser | Crist Instrument | 5-RLD-D1 |
