# Supplementary material for: Neural population dynamics and temporal context cells in macaque medial parietal cortex support temporal order memory
Source: PLoS Biol. 2026 Apr 17;24(4):e3003759. doi: 10.1371/journal.pbio.3003759 (PMC13108878; doi:10.1371/journal.pbio.3003759)
Supplement: Attachment — Submitted filename: Response to Reviewers.pdf [file pbio.3003759.s009.pdf]

Overview:

We are grateful for the time and effort that the Editors and the reviewers devoted to evaluating our manuscript entitled *"Neural signatures for temporal order memory in the macaque medial posterior parietal cortex"* (previous manuscript number: **PBIOLOGY-D-25-00280R1**). We found the reviewers' critiques extremely constructive and have undertaken a substantial re-analysis of the data to address the central concerns raised. Specifically, several reviewer comments overlapped conceptually. To avoid redundancy, in this "*Response to Reviewers*" letter, we describe our GLM framework and control analyses once in detail and refer back to these sections where relevant.

Reviewer #1:

The study employed in vivo multi-unit electrophysiology in macaques performing a temporal-order judgment (TOJ) task using naturalistic videos. Neuronal activity in the medial posterior parietal cortex (mPPC) was recorded during encoding (video viewing) and retrieval (TOJ decisions). Temporal context cells, identified via ex-Gaussian model fitting, encoded temporal information during encoding, with diverse relaxation times forming a spectrum of temporal representations. Linear discriminant analysis (LDA) demonstrated that neuronal ensembles could decode temporal progression within videos. During retrieval, TOJ cells exhibited activity linked to decision accuracy, and spike synchrony (measured via SPIKE-distance) increased prior to correct responses. Encoding-retrieval neural pattern similarity (Mahalanobis distance) correlated with performance, and control experiments ruled out eye movement confounds. A subset of temporal context cells showed context-dependent responses in segmented videos, suggesting adaptability to event boundaries.

Overall, this is a very interesting and important work. The authors used an approach that combines naturalistic video stimuli with electrophysiology, bridging ecological validity and neural mechanistic insights. They identified temporal context cells in mPPC and linked their activity to memory retrieval, expanding understanding beyond hippocampal-prefrontal frameworks. They also included rigorous controls, including eye-tracking. I only have a few relatively minor comments.

Response: We thank the reviewer for their time and effort in reviewing our work. In the following, we addressed their comments by adding new analyses and detailed clarification, as stipulated in this response letter and in the revised manuscript.

1. First, it is not very clear to me how the temporal context cells were identified. The authors might want to clarify the rationales in more detail before the results. Did the authors suggest that the temporal context cells should respond to the onset of the video, and the various relaxation times serve as a time meter to label the passage of time? The paper needs to clarify how their "spectrum" of relaxation times directly enables temporal order judgments. For the current study, since it was always the 5th, 90th, 95th, and 190th frame selected for TOJ, shall we expect that only a few cells label these frames and contribute to TOJ?

Response: We thank the reviewer for raising this important point and agree that the rationale for identifying temporal context cells and their relevance to TOJ warrants clarification. Following the framework established by Bright et al. (2020), temporal context cells are defined by their transient

responses aligned to a salient event boundary, followed by exponentially decaying activity with heterogeneous relaxation time constants. In that work, the onset of an event (e.g., stimulus onset) serves as a temporal anchor, and the population-level spectrum of decay constants provides a continuously evolving representation of elapsed time since that event. In our paradigm, the onset of the cinematic episode plays an analogous role as the temporal reference point. We do not assume that individual cells explicitly "label" specific frames. Rather, elapsed time is implicitly encoded in the distributed population state, determined by the relative activity across cells with different relaxation time constants.

Temporal order judgments are therefore supported by comparing population states, whose similarity reflects the relative temporal distance between moments within the episode. Importantly, although TOJ probes were drawn from fixed frame indices (e.g., 5th, 90th, 95th, and 180th frames), this does not imply that only a small subset of cells contributes to TOJ. Instead, all temporal context cells contribute continuously, with different subsets dominating at different time scales. The discriminability of temporal order arises from population-level representational geometry, not from frame-specific or event-specific coding by individual neurons. We revised the manuscript according to the explanations given above to more clearly articulate (i) the role of episode onset as a temporal anchor and (ii) how a spectrum of relaxation times enables temporal order judgments through distributed population dynamics rather than discrete time stamping. See **pp. 12-13** in the revised manuscript.

2. Second, the LDA based on the ensembles of neuronal response makes more sense to me. I have two questions. First, did the decoding accuracy correlate with behavioral performance? Second, could you do some representational similarity analysis to show how fast the temporal context drifts with time?

Response: We thank the reviewer for their positive assessment of the LDA approach and for suggesting two additional analyses. Following these suggestions, we performed both analyses and obtained results that were not included in the original submission. These new results are now incorporated into the revised manuscript.

First, we examined whether LDA decoding accuracy was related to behavioral performance in the temporal order judgment task. We performed linear discriminant analysis separately for each recording session (n = 63) using a standardized 5-fold cross-validation procedure (see also our response to Reviewer 3, Major Comment 8), yielding one decoding accuracy value per session. We then assessed the relationship between decoding accuracy and behavioral performance across sessions and found a significant positive correlation ($r = 0.26$, $p = 0.039$; Figure R1 below and **updated Figure 5H** in the revised manuscript). This result indicates that session-to-session variability in ensemble-level neural discriminability during encoding predicts subsequent TOJ performance during retrieval.

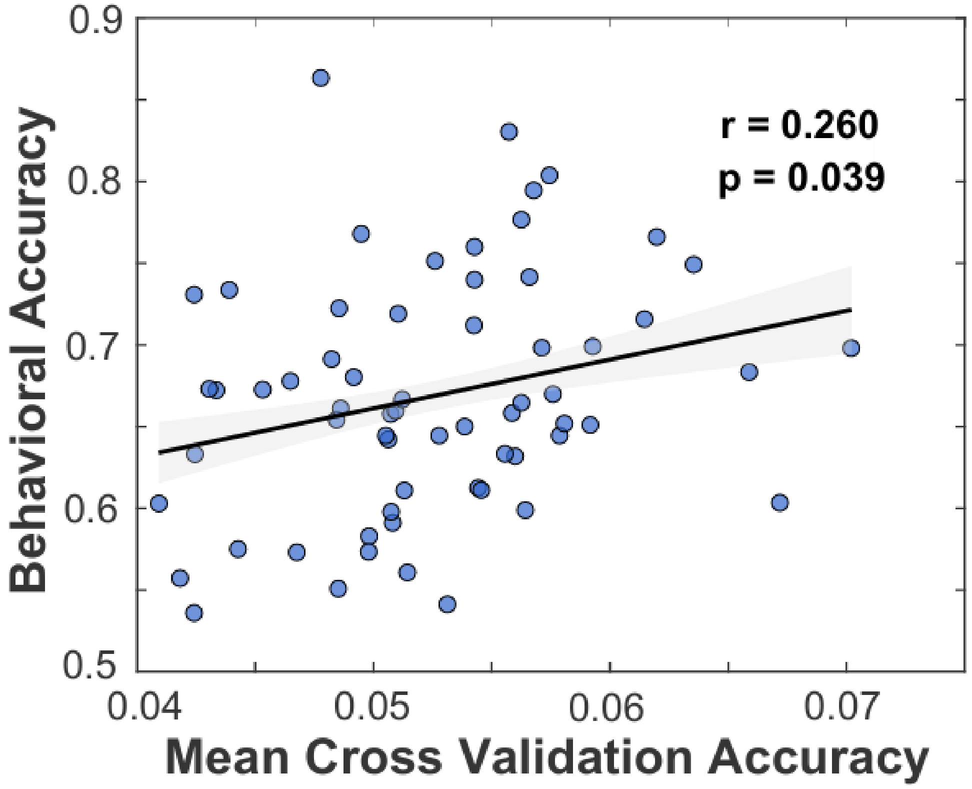

Figure R1. Relationship between LDA decoding accuracy during encoding and subsequent temporal order judgment performance across recording sessions. Each dot represents one session collapsed across monkeys (n = 63). This analysis is now included in **updated Figure 5H** of the revised manuscript.

Second, we examined how temporal context drifts over time during encoding using representational similarity analysis (RSA). We binned spikes into 250-ms windows, z-scored activity across neurons, and computed Pearson correlations between all pairs of time bins to form representational similarity matrices. Similarity was highest along the diagonal and decayed with increasing temporal separation, quantifying the rate at which population activity drifted over time during movie watching (Figure R2 below). This decay profile quantifies the rate at which population-level neural representations drift over time during encoding, consistent with a gradually evolving temporal context signal. These results have been added to the revised manuscript (**p. 8; updated Figure 5G**).

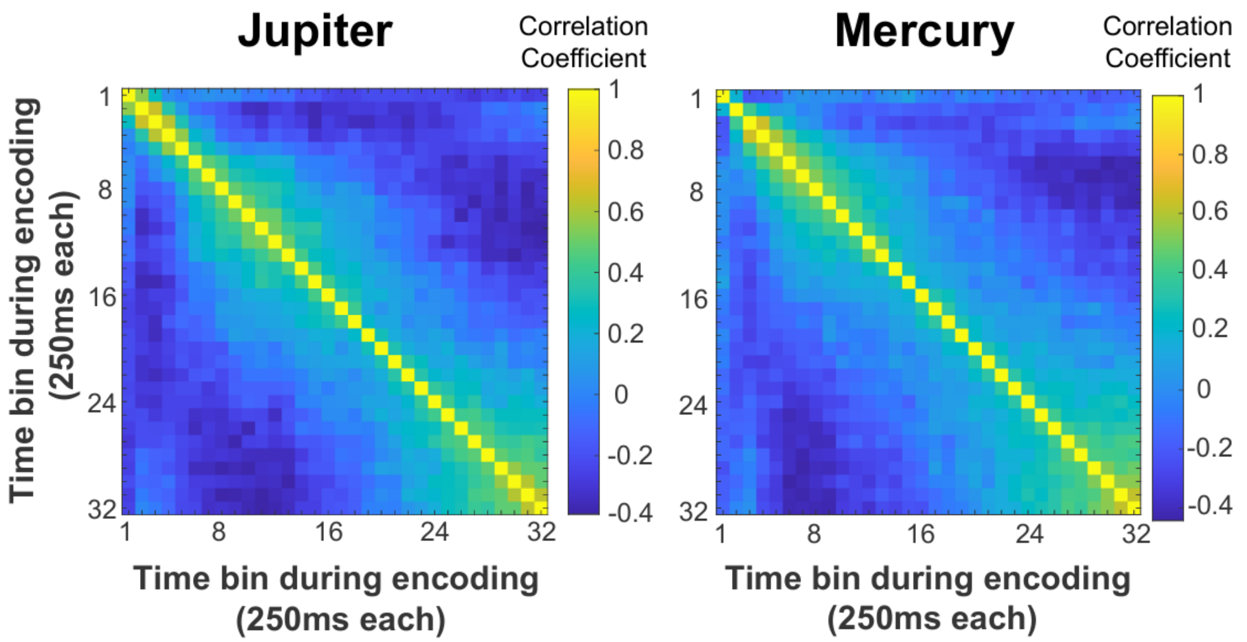

Figure R2. Representational dissimilarity matrices showing Pearson correlation coefficients neural population activity across all pairs of time bins during movie watching for each monkey.

3. Third, the spike synchrony (measured via SPIKE-distance) result is interesting. I am wondering if the authors could provide more discussion on the underlying mechanism? If I understand the result correctly, they found the SPIKE-distance was lower for the correct than incorrect trials. However, for immediate and delayed comparison, the behavioral performance was better in the delayed condition, whereas the synchrony level is consistently higher for the immediate condition than for the delayed condition.

Response: We thank the reviewer for this insightful question. The apparent dissociation between behavioral performance and overall spike synchrony across conditions reflects a distinction between within-condition trial-by-trial effects and across-condition task states. Within each condition, lower SPIKE-distance (i.e., higher synchrony) was associated with correct compared to incorrect trials, suggesting that coordinated population activity supports successful temporal order judgments under a fixed task regime. Importantly, regarding the SPIKE-distance for the correct vs. incorrect trials comparison, this correctness synchrony effect is only manifested about 500 ms or longer *preceding* the animals' final decision (**updated Figure 3 A-B** bottom panels).

Across conditions, however, the immediate and delayed conditions differ in the degree to which behavior relies on externally driven sensory input versus internally maintained temporal context. The immediate condition (i.e., no retention between end of the movie and onset of TOJ images) involves stronger sensory drive and shorter retention, which likely induces higher overall spike synchrony due to shared inputs and tighter temporal alignment across neurons. In contrast, the delayed condition places greater demands on internally generated temporal context representations, which may be supported by more distributed and less synchronized population activity. Moreover, there is a further notable difference from within-condition effect (correct vs. error responses). The pattern for immediate vs. delayed difference is only seen when the comparison is time–locked to the onset of the TOJ images at about 200 ms onwards (**updated Figure 3 C-D**, upper panels) but

not towards the moment of responding (**updated Figure 3 C-D**, bottom panels). The former refers to the period right after the display of the two probe images (onset time-locked to 0 s), whereas the latter refers to the period preceding the animals' memory decision (offset time-locked to 0 s).

Thus, higher global synchrony does not necessarily imply better performance across task conditions, especially when such effects are not seen upon/towards memory decisions. Rather, spike synchrony reflects the underlying network state and coding regime and not necessarily performance (especially during onset of TOJ images) (Harris & Thiele, 2011), whereas successful temporal order judgments depend on the stability and fidelity of temporal context representations.

We have added discussion to clarify this distinction in the revised manuscript (see **pp. 6, 11-12;** and our response to Reviewer 3, Major Comments 3 b-d). Note also our attempt to dissociate sensory-driven from retrieval-related activity with new analyses (see our response to Reviewer 2, Major Comment 2).

Minor Points for Clarification/Correction:
1. Abbreviations: Define all terms (e.g., TOJ, mPPC) at first mention.

Response: We have all abbreviations defined at their first mention.

2. Statistical Details: Specify parameters for permutation tests (e.g., number of shuffles) and correct for multiple comparisons in SPIKE-distance analyses.

Response: In response to the reviewer's request, we have specified the parameters of the permutation tests and applied correction for multiple comparisons in the SPIKE-distance analyses. We re-ran all relevant statistical analyses using a stricter, nonparametric permutation-based approach. Specifically, statistical significance of group differences was assessed using 1,000 random permutations. In each permutation, the entire dataset was randomly shuffled and reassigned into two groups while preserving the original group sizes, thereby eliminating any systematic group structure. Independent-samples t-statistics were computed for each shuffled dataset to generate a null distribution of t-values under the assumption of no true group differences.

The significance of the observed t-statistics was evaluated using a two-tailed permutation test, with the p-value defined as the proportion of permuted t-values whose absolute values equaled or exceeded that of the observed statistics. To correct for multiple comparisons in the SPIKE-distance analyses, all p-values were further adjusted using the false discovery rate (FDR) procedure (MATLAB *mafdr* function; corrected threshold $p < 0.01$). These methodological details and the updated results have been added to the revised manuscript (**p. 21**).

3. Figure Labels: Ensure all panels in Figures 1-8 are explicitly referenced in the main text.

Response: Based on reviewers' comments we have modified all the original figures and ensured we make correct references to them in the revised manuscript. We have followed Reviewer 3's suggestion (Minor Comment 7) to focus more on the retrieval /TOJ results to highlight the novelty of the work.

4. Data Availability: The GitHub link for code/data is incomplete; provide a working URL or accession number

Response: We have deposited code and data on Dryad (link provided in the revised manuscript; will be activated upon acceptance).

References:

Bright IM, Meister MLR, Cruzado NA, Tiganj Z, Buffalo EA, Howard MW. A temporal record of the past with a spectrum of time constants in the monkey entorhinal cortex. Proceedings of the National Academy of Sciences of the United States of America. 2020;117(33):20274-20283.

Harris, K. D. & Thiele, A. Cortical state and attention. Nat. Rev. Neurosci. 2011; 12, 509–523.

Reviewer #2

Reviewer #2 (Yuji Naya): The paper presented electrophysiological data from the medial posterior parietal cortex (mPPC) of macaques during the temporal-order judgement task using video-clips as a sample. The authors defined the temporal context cells by referring to preceding literature by Bright et al., 2020 (ref 26). These neurons could show the passage of time as a population. In addition to the encoding, the present paper presented responses during the temporal order judgment period, in which retrieval may happen, and showed many neurons with significant responses (447 out of 676 recorded neurons). In addition to single-neuron based analysis, The authors showed retrieval effect by examining spike-synchrony among recorded neurons, suggesting higher synchrony in correct trials. Taken together, the present paper suggests involvements of the mPPC in both encoding and retrieval of temporal order memories. Temporal order memory is an important cognitive function for our daily life and its relevance to the PPC sounds exciting. I agree with the authors that the investigation of temporal order memory in the mPPC is timely.
However, I have several concerns particularly in the analyses.

Response: We thank the reviewer for his time and effort in reviewing our work. In the following, we took their comments into careful consideration and made significant changes to the manuscript on both conceptual and data analytical levels. The new analyses and many conceptual discussions in light of the extant literature significantly help improve the manuscript. We reported them in the revised manuscript as well as have them highlighted in this response letter.

Main concerns

1) I don't deny a possible involvement of mPPC in encoding temporal contexts like the primate entorhinal cortex, which might contribute to generation of time signal in other brain area (e.g., hippocampus) as the authors discussed (Page 8, last paragraph). However, each temporal context neuron might show just a visual response even thou the visual response could contribute to decoding of the time passage. I may expect that we could decode the time passage during the video in many of brain areas with visual responses. I wonder if these areas also signal temporal context. Please make this point clear.

Response: We thank the reviewer for this important conceptual point and fully agree that decodability of elapsed time alone does not imply the presence of a temporal context representation. Indeed, as the reviewer correctly notes, visually responsive neurons in many cortical areas may carry information that allows elapsed time during a video to be decoded, simply due to stimulus-driven dynamics. Our use of the term *temporal context* follows the framework developed in prior work by Howard and colleagues and is also consistent with the reviewer's influential studies on associative and contextual coding in the primate medial temporal lobe and connected cortical areas (e.g., Naya et al., 2001, 2003; Naya & Suzuki, 2011). In this framework, temporal context is not defined by time decodability or visual responsiveness *per se*, but by a slowly evolving population state that links temporally separated events and supports temporal order and associative memory.

Consistent with this distinction, nonhuman primate studies show that strong stimulus-driven responses in ventral visual cortex (e.g., area TE) do not necessarily entail temporal-order or 'when' coding, whereas medial temporal lobe structures more directly support item–time integration and temporal order memory (e.g., Naya & Suzuki, 2011; Naya et al., 2017). Accordingly, we do not

interpret our decoding results alone as evidence for temporal context but rather emphasize convergent evidence that population activity in mPPC evolves gradually across an episode, predicts subsequent temporal order judgment performance, and reinstates encoding-related population states during retrieval.

Importantly, we do not claim that mPPC uniquely or independently encodes temporal context. Instead, temporal context signals may emerge in cortical regions that integrate sensory information over extended timescales and interact with medial temporal lobe memory systems. In this sense, our findings are intended to complement, rather than replace, existing accounts of temporal-order memory grounded in medial temporal lobe circuitry (e.g., Naya et al., 2001; Naya et al., 2003).

We revised the manuscript to clarify that (i) time decodability alone is insufficient to establish temporal context, (ii) visually driven responses may contribute to temporal signals in many brain areas, and (iii) our interpretation of mPPC emphasizes its role in supporting temporally extended, memory-relevant population dynamics engaged during TOJ, rather than claiming a unique or stimulus-independent locus of temporal context coding. See **pp. 12-14** in the revised manuscript.

2) The present study detected many TOJ cells by GLM including trial events as regressors. The authors suggest retrieval-related responses (Page 5). However, the main force of responsiveness during the temporal-order judgment might be presentation of target stimuli, which may elicit either excitation or inhibition. How much of responses can be explained by retrieval process out of the total response change, which may include visual responses to target stimuli?

We thank the reviewer for raising this important point. We agree that neural responses during the TOJ period may reflect a mixture of processes, including sensory responses to the presentation of the target stimuli, decision-related processes, and memory retrieval. Our goal in the present study is not to claim that TOJ cells are purely retrieval-specific, but rather that their activity during the TOJ period is consistent with engagement of retrieval-related processes. In our main GLM, trial events—including target stimulus presentation—were explicitly modeled, allowing us to identify neurons whose activity was modulated during the TOJ epoch over and above baseline. We acknowledge that part of the observed response change may be driven by visual input.

To address the concern that TOJ-related responses may be driven by visual stimulation over and above memory retrieval, we now additionally ran a control GLM that explicitly models and removes variance associated with visual onset responses, allowing retrieval- and decision-related modulation to be assessed more conservatively. We extended the original GLM by decomposing the TOJ period into **visual-onset–locked**, **post-onset retrieval-related**, and **response-locked motor** components: (i) Target visual onset regressor is time-locked to the onset of each TOJ probe stimuli and is modeled as a brief impulse or short boxcar (e.g., 0–200 ms; cf. neuronal latencies of visual response in Naya et al. 2001 and our present revised Figure 3 for insights into this hypothesized time course), capturing early sensory/visual driven responses; this regressor is used across all TOJ trials to capture shared visual drive. (ii) Post-onset TOJ regressor (retrieval/decision window) is time locked after a short latency following stimulus onset (e.g., ≥ 200 ms onwards till 200 ms before TOJ response); this regressor is designed to capture sustained activity associated with retrieval, comparison, and memory decision formation, after the initial visual transient has

been accounted for. (iii) Response-locked motor-related regressor is time locked to 200 ms before animal's response execution till response; this regressor is designed to capture some of the activity related to the hand movement.

If TOJ-related activity were primarily driven by visual input, significant effects should be absorbed by the visual-onset regressor, leaving little residual modulation. Conversely, if retrieval and decision processes contribute meaningfully, significant activity should remain in the post-onset TOJ regressor, particularly in relation to temporal-order variables. This would support the interpretation that TOJ-related responses are not reducible to simple sensory responses. We found that significant TOJ-decision modulation remained after accounting for early sensory responses (as well as putative motor responses).

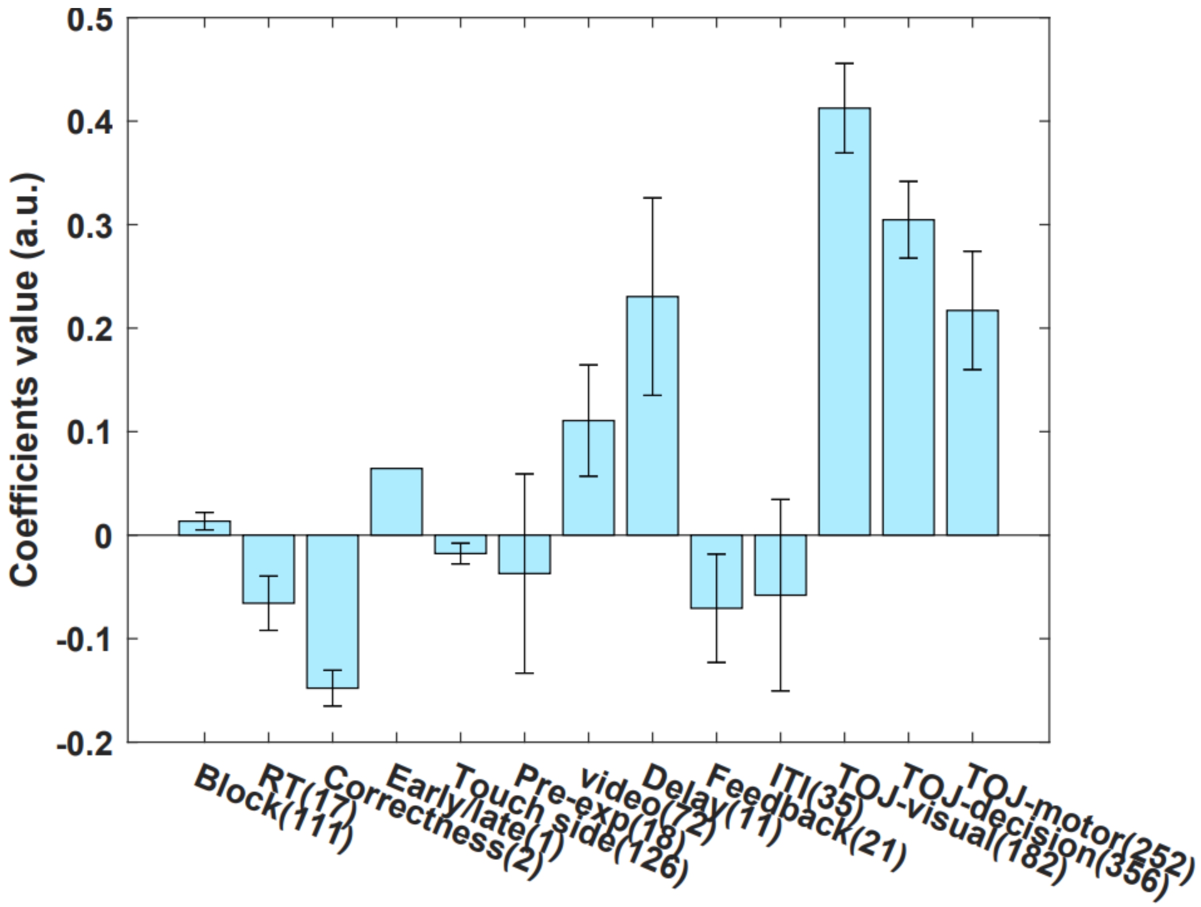

Figure R3. GLM results including sub-components for the TOJ period. Numerals in bracket indicate the number of neurons that are statistically modulated by specific variables.

In sum, we decomposed the TOJ epoch into an early visual-onset window (0–200 ms post-probe onset), a post-onset retrieval/comparison window (200 ms post-onset until 200 ms before response), and a response-locked motor window (−200 ms to response), allowing TOJ-related modulation to be assessed after accounting for early sensory and motor-related responses. We modified the Methods section, provided the corresponding model formulae, added these results in the revised manuscript, see Figure R3, **updated Figure 2B**, and **pp. 4-5, 20**.

We would also like to reiterate that our interpretation of retrieval-related activity is population-level (also see results on neuronal synchrony section), rather than attributing retrieval specificity to individual neurons. The smaller but reliable modulation observed during the TOJ period—prior to response execution and reward delivery (see also our response to Reviewer 2, Minor Comment 1)—occurs at the time when temporal context representations are expected to be accessed and compared. We therefore interpret this activity as reflecting retrieval and decision processes that are engaged by, but not fully separable from, stimulus presentation in this task, see **p. 5** in the revised manuscript.

3) "72 (~10.7%) neurons differed in their firing rates between correct response and incorrect response (one-tailed t-test, all Ps<0.05)." (Page 6, Lines 1-2)
In this analysis, 'two-tailed' would be appropriate because you examined the difference rather than just only increase from incorrect to correct (or only decrease). Please show the corrected number of neurons with significant difference and compare it with its expected distribution statistically (e.g., two binominal test or chi-square test).

Response: To assess whether individual neurons exhibited firing rate differences between correct and incorrect trials during the TOJ period, we compared firing rates using two-tailed t-tests for each neuron. This analysis identified 59 of 676 neurons (8.7%) with significant differences between correct and incorrect trials (Jupiter: 23/401 neurons; Mercury: 36/275 neurons; all Ps < 0.05). To determine whether this proportion exceeded chance expectations, we performed binomial tests assuming a nominal significance level of 0.05. When pooling data across monkeys, the observed number of significant neurons exceeded chance levels (P < 0.001). However, when analyzed separately, this effect was driven primarily by one animal (Mercury), whereas the number of significant neurons in the other animal (Jupiter) did not differ from chance.

After correcting the analysis to two-tailed tests and evaluating chance-level expectations, the firing-rate differences between correct and incorrect trials are limited, variable across animals, and not specific to TOJ neurons (see our response to Reviewer 3, Major Comment 3a). Given the corrected neuron counts and the absence of neuron-category specificity, we have moved a part of this discussion to the Supplementary Materials (**Supplemental Text S1**) and revised the main Results to de-emphasize this effect. We believe this change better reflects the secondary nature of correctness-related firing rate modulation relative to the main findings, which focus on decision-related dynamics and population-level coordination during temporal order judgment.

4) "the Mahalanobis distance was consistently smaller during correct trials" (Page 6, last line) This might be because responses during the early of the encoding period were generally stronger and contributed to the population vector of encoding responses more and the monkeys might watched the correct target, which was presented during the early of the encoding period, in correct trials. In short, this result might be explained just by visual responses rather than memory effect. Please address this point.

Response: We thank the reviewer for raising this alternative explanation. We note that this concern may stem from a misunderstanding of the task design: correct trials are not selectively associated with early-encoding frames. Probe pairs were sampled from either the first half (0–4 s; i.e., 5th vs.

90th frame) or the second half (4–8 s; i.e., 95th vs. 180th frame) of the video, and both trial types occurred throughout the experiment. Consequently, many correct trials involve probe frames from the latter half of the video (i.e., the 95th frame), which is inconsistent with an account based solely on stronger visual stimulation during early part of the encoding period.

Nonetheless, our task design allows this possibility to be directly tested. In the retrieval phase, target–foil pairs were drawn either from the first half (0–4 s; i.e., 5th vs. 90th frame) or the second half (4–8 s; i.e., 95th vs. 180th frame) of the encoded videos, with these trial types equally represented. To directly test whether the reduced encoding–retrieval Mahalanobis distance observed in correct trials could be explained by stronger visual responses early during encoding, we reanalyzed Mahalanobis distance separately using firing-rate vectors derived from the first half (0–4 s) or the second half (4–8 s) of the encoded video. We then fit a linear mixed-effects model with trial correctness, encoding video half, and delay condition as fixed effects, and monkey identity as a random effect. This analysis allowed us to assess whether encoding–retrieval similarity depended on memory accuracy independently of when during encoding the population activity was sampled.

MDist ~ Correctness * VideoHalf * Delay + (1 | Monkey)

Trial correctness exerted a robust effect, with correct trials showing significantly smaller Mahalanobis distances than incorrect trials ($p < 0.001$). In contrast, video half had no significant main effect ($p = 0.24$), nor did it interact with correctness ($p = 0.23$), indicating that encoding–retrieval similarity was not systematically influenced by whether probes originated from the early or late portion of the video. These results rule out a trivial explanation based on stronger early visual responses and support the interpretation that reduced Mahalanobis distance reflects memory-related reinstatement rather than sensory-driven effects. We have added this control analysis and clarification to the revised manuscript (**pp. 9, 23**).

5) "We identified the TOJ cells that are also classified as temporal context cells, observing that ~70.3% (78 out of 111 cells) of the temporal context cells belonged to the TOJ cells class (Figure 6C-D). A chi-square test revealed that this proportion is statistically significantly larger than what would be expected by chance (χ2(1) = 91.203, P < 10-5)." (Page 7, 2nd paragraph). Please present the expected number of neurons showing both TOJ and temporal context effects in the rest section. In my understanding, if to be a temporal context cell and TOJ cell is independent, it would be 111×447/676 = 73.4. Is this number significantly different from the real (n = 78)?

Response: We thank the reviewer for pointing out the need to explicitly report the expected number of neurons showing both TOJ and temporal context effects and for providing the correct intuition under the assumption of independence. In the revised analysis, we explicitly tested whether the observed overlap between TOJ cells and temporal context cells (TCCs) exceeded chance expectations. Given that 111 of 676 neurons were classified as temporal context cells and 461 of 676 neurons were classified as TOJ cells, the expected number of neurons belonging to both categories under independence is $(461 \times 111) / 676 = 75.7$. The observed number of neurons showing both TOJ and temporal context effects was 78, which does not significantly differ from this expected value ($\chi^2(1) = 0.084$, $P = 0.772$). In addition, the proportion of temporal context cells

did not differ significantly between TOJ and non-TOJ neuron populations ($\chi^2(1) = 0.162$, $P = 0.688$). Together, these results indicate that, in the present task context, temporal context cells in posterior parietal cortex are not preferentially enriched within the TOJ neuron population but instead occur independently of TOJ classification (Figure R4). Based on these findings, we have revised the Results section to remove the previous claim of above-chance overlap (**pp. 9-10**). We also noted that the number of TOJ neurons is 461 (not 447 as stated in the original submission); this counting error has been corrected throughout the revised manuscript.

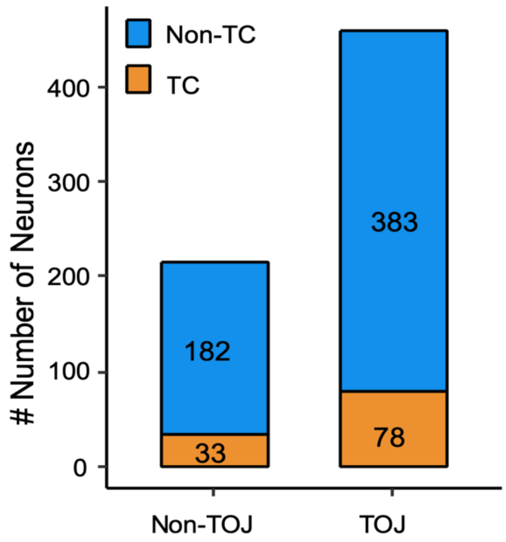

Figure R4. Distribution of recorded neurons classified as temporal order judgment (TOJ) cells and temporal context cells (TCCs). The observed overlap does not differ from chance expectations, indicating that temporal context cells are not preferentially enriched within the TOJ neuron population.

Minor concerns

1) In Figure 5A&B, the most striking part might be the difference after TOJ offset. Please present a possible reason why the big difference happened and explain why much smaller difference during the TOJ period is still important.

Response:

We thank the reviewer for this insightful observation. We agree that the difference following TOJ offset is visually prominent. Importantly, this post-offset difference is observed in the correct versus incorrect contrast but not in the immediate versus delayed contrast (see **updated Figure 3C–D**), suggesting that it reflects processes other than temporal order computation per se. A likely explanation is that neural activity after TOJ offset reflects post-decisional processes, including commitment to the decision, motor execution, and updating or disengagement of the active memory state once a judgment has been completed. In addition, correct trials were consistently followed by water reward delivery, which is expected to evoke strong reward-related responses such as outcome evaluation and consummatory signals. These post-decisional and reward-related processes likely amplify neural differences after TOJ offset and are therefore not specific to

temporal order processing. In contrast, the smaller but reliable difference observed during the TOJ period is critical because it occurs while the temporal order judgment is actively being computed—prior to response execution and reward delivery. This time window therefore more directly isolates the mnemonic and decision-related processes underlying temporal comparison. Although the effect size during TOJ is smaller, its temporal alignment with the cognitive operation of interest makes it central to our interpretation. We have clarified this distinction in the revised manuscript to explicitly explain why the during-TOJ effect, despite its smaller magnitude, provides the most direct evidence for neural mechanisms supporting temporal order computation (**pp. 6–7**).

2) "The synchrony level is consistently higher for the delayed condition compared to the immediate condition both time-locked to TOJ onset (Figure 5C-D top) or at monkeys' responses (Figure 5C-D bottom). Since memory traces for trials without a retention delay ought to be stronger than those with a longer retention delay, these data imply that a high synchronization among spike trains is a neural proxy for memory strength." (Page 6, 2nd paragraph)
Is this a confusion between "synchronous level" and "SPIKE-distance values"? If yes, please correct it.

Response: We thank the reviewer for pointing out this confusion. The reviewer is correct that this statement resulted from a misinterpretation between synchrony level and SPIKE-distance values. In our analysis, lower SPIKE-distance values correspond to higher spike train synchrony. The original sentence incorrectly described the direction of the effect by conflating these two measures. We have corrected the text to accurately reflect the results shown in **updated Figure 3C–D**, clarifying that spike train synchrony is higher (i.e., SPIKE-distance values are lower) in the immediate condition compared to the delayed condition. The revised wording now aligns with both the quantitative definition of the synchrony metric and the empirical results. This correction is purely terminological and does not affect the analyses, figures, or conclusions of the study. The manuscript has been revised accordingly (**p. 6**); see also our response to Reviewer 3, Minor comment 5.

3) Why did the authors use two video-clips for encoding in the Experiment 2? The result in the Experiment 2 may not be appropriate as a control of the experiment 1 because not only the task difficulty but also the stimulus conditions during the TOJ period, which you examined, differ essentially. Please present a rational to use the Experiment 2 clearly in the text.

Response:
We thank the reviewer for raising this important concern. We agree that Experiment 2 differs from experiment 1 not only in task difficulty but also in stimulus structure during the TOJ period, and therefore it should not be regarded as direct control or replication of experiment 1. The primary motivation for Experiment 2 was to address a specific potential confound in experiment 1, namely the contribution of eye-movement–related interference and continuous visual flow associated with viewing a single uninterrupted naturalistic video. In experiment 1, the continuous 8-s video may elicit smooth pursuit, systematic scan paths, and temporally structured eye movements that could introduce additional temporal correlations in neural activity unrelated to temporal context memory per se. To mitigate this concern, in Experiment 2 we trained a separate monkey and used two discrete 4-s video clips, which disrupt prolonged continuous motion and narrative flow. Together with simultaneous eye-tracking and regression analyses of saccade, fixation, and scan-path

variables, this design allowed us to assess whether TOJ-related neural activity could be explained by oculomotor behavior. Importantly, we do not interpret Experiment 2 as providing additional or independent evidence supporting the main conclusions of experiment 1. Rather, it serves as a control analysis to rule out eye-movement-related confounds and to verify that the observed TOJ-related neural activity cannot be accounted for by fixation patterns, saccades, or scan paths. We have modified parts of the abstract, methods, and main text to reflect the limits of Experiment 2 and to clarify this rationale explicitly in the revised manuscript to avoid overinterpretation, see **pp. 2, 10, 18** in the revised manuscript and our response to Reviewer 3, Major Comment 5 and Minor Comment 7.

References:

Naya, Y., Yoshida, M., & Miyashita, Y. (2001). Backward spreading of memory retrieval signal in the primate temporal cortex. Science, 291(5504), 661–664.

Naya, Y., Yoshida, M., & Miyashita, Y. (2003). Forward processing of long-term associative memory in monkey inferotemporal cortex. Journal of Neuroscience, 23(7), 2861–2871.

Naya, Y. & Suzuki, W. A. (2011). Integrating what and when across the primate medial temporal lobe. Science, 333(6043), 773–776.

Naya, Y., Chen, H., Yang, C., & Suzuki, W. A. (2017). Contributions of primate prefrontal cortex and medial temporal lobe to temporal-order memory. Proceedings of the National Academy of Sciences of the United States of America, 114(51), 13555–13560.

Reviewer #3:

Reviewer #3: Title:
Neural signatures for temporal order memory in the macaque medial posterior parietal cortex

Overall evaluation:
In this manuscript, the authors displayed behavioral and electrophysiological data collected from the medial posterior parietal cortex (mPPC) of 3 monkeys (but majority of analyses were done using 2), who performed temporal order discrimination tasks using frames of short (8s, or 4s for one monkey) videos. The authors found that mPPC neuronal firing rates could be used to decode time within the encoding period, and that certain mPPC neurons' activities were significantly correlated with temporal context and/or temporal order judgment (TOJ). TOJ-associated neurons had different firing/synchrony profiles for correct versus incorrect trials during the TOJ time period, and appeared to have similar populational activity patterns between encoding and TOJ (where memory retrieval is required) at the single-trial level. Taken together, these results do offer some support for the authors' claim that mPPC neuronal ensembles may be involved in TOJ and the memory retrieval it necessitates. However, current results may be insufficient to illustrate the potential role of these neurons in processing ordinal information/sequential stimuli. Additional evidence against more mundane explanations of the current observations may also be required to make the analyses more robust.

Response: We thank the reviewer for the thoughtful and detailed assessment of our manuscript. In response to these comments, we have undertaken additional analyses, corrected and clarified statistical procedures, and revised the presentation and interpretation of several results to improve transparency and rigor. In particular, we clarified the definition and identification of TOJ neurons using an explicit Poisson GLM framework, implemented additional controls to dissociate sensory, mnemonic, and motor-related components of TOJ-related activity, re-evaluated firing rate and synchrony effects using appropriate statistical tests, and revised or de-emphasized analyses that were exploratory or insufficiently supported. We believe these changes substantially strengthen the manuscript and address the reviewer’s concerns.

Major issues:
1. On experiment design: the task frame locations appear to be identical across trials/blocks (5/90, or 95/180); why not vary the chosen frames as randomly assigned within a video segment? With identical frame locations, the risk is that monkeys could rely on a report strategy on a rote memory basis (i.e. always touching frames 5/95) instead of using TOJ.

Response: We thank the reviewer for this insightful comment regarding the use of fixed frame locations rather than randomly selected frames.

Our use of fixed frame locations was motivated by both theoretical and practical considerations. Prior behavioral work demonstrated that macaques perform temporal order judgments using a forward replay–like mechanism, evidenced by systematic relationships between reaction time and the temporal position of the chosen frame (Zuo et al., 2020). To directly test the neural correlates of this process, it was essential to tightly control the temporal structure of the task. Accordingly, we fixed frame locations so that temporal distance was held constant across conditions (TD = 85 frames), allowing neural comparisons to isolate temporal-order processes rather than variability in

temporal separation or stimulus similarity (Brown et al., 2007). Introducing randomly varying frame positions would have added uncontrolled variability to temporal similarity and delayed neural comparisons, thereby reducing interpretability of population-level temporal signals. Moreover, we believe it is unlikely that monkeys could solve the task via a rote or frame-counting strategy. Across sessions, animals viewed a large number of unique, naturalistic video stimuli, each containing rich and continuously changing visual content. Moreover, the minimal temporal separation between the 90th and 95th frames makes counting-based strategies infeasible. Critically, the 95th frame served as the target while the 90th frame served as a foil—opposite to the learned TOJ rule—further preventing reliance on fixed-location strategies.

Taken together, the behavioral constraints, stimulus diversity, and neural evidence support the interpretation that monkeys relied on temporal order judgments rather than rote memorization. We therefore believe that the fixed-frame design was appropriate for addressing the central theoretical questions of this study. These important considerations are now added in the revised manuscript, see **p. 18**; please refer also to our response to Reviewer 3, Major Comments 6.

2. Although the authors identified TOJ neurons, the GLM-based approach can only demonstrate that the activity of these neurons increased following the presentation of TOJ stimuli. There are numerous other possibilities that could explain this task-relevant response, such as responses to stimulus onset, attention arousal, or motor preparation, among others. These factors may also differ between correct and incorrect trials. Consequently, merely comparing the differences in firing rate and spike synchronization between correct and incorrect trials is inadequate to determine whether these neurons are truly involved in TOJ processing and what specific cognitive processes they encode within TOJ. Here are some possible suggestions: 1) test the correlation between the TOJ neuron activities and the TOJ event in error trials. If TOJ neurons are indeed involved in order encoding, such a correlation should be attenuated when the animals make errors; 2) test the correlation between the TOJ neuron activities and RTs.

Response: We thank the reviewer for these thoughtful comments and agree that increased firing following TOJ stimulus presentation alone does not uniquely establish TOJ-specific processing, as such activity could reflect stimulus onset, arousal, attention, or motor preparation. To more directly test whether TOJ neurons are specifically linked to temporal order judgment rather than general task engagement, we conducted additional analyses motivated by the reviewer's suggestions. Specifically, we examined the relationship between neuronal activity and behavioral response time (RT), which provides a continuous measure of decision formation and is widely used to dissociate decision-related signals from sensory- or motor-related responses. We assessed whether TOJ neurons exhibit stronger trial-by-trial coupling to behavioral performance than non-TOJ neurons, and whether this relationship differs between correct and incorrect trials. For each neuron, we computed the trial-wise slope of the RT–firing rate relationship separately for correct and incorrect trials (that is, *trial-wise RT ~ firing rate slope*). These slopes were entered into a linear mixed-effects model with neuron category (TOJ vs. non-TOJ), trial outcome (correct vs. incorrect), and their interaction as fixed effects, and animal identity as a random effect. The results revealed a robust main effect of neuron category: TOJ neurons showed a significantly stronger negative RT–firing rate relationship than non-TOJ neurons (mean difference = −0.593, $p = 3.29 \times 10^{-7}$; see Figure R5 and **updated Figure 2D** in the revised manuscript). This indicates that higher firing

rates in TOJ neurons are associated with faster temporal order judgments, supporting their specific involvement in TOJ-related decision processes rather than nonspecific task factors.

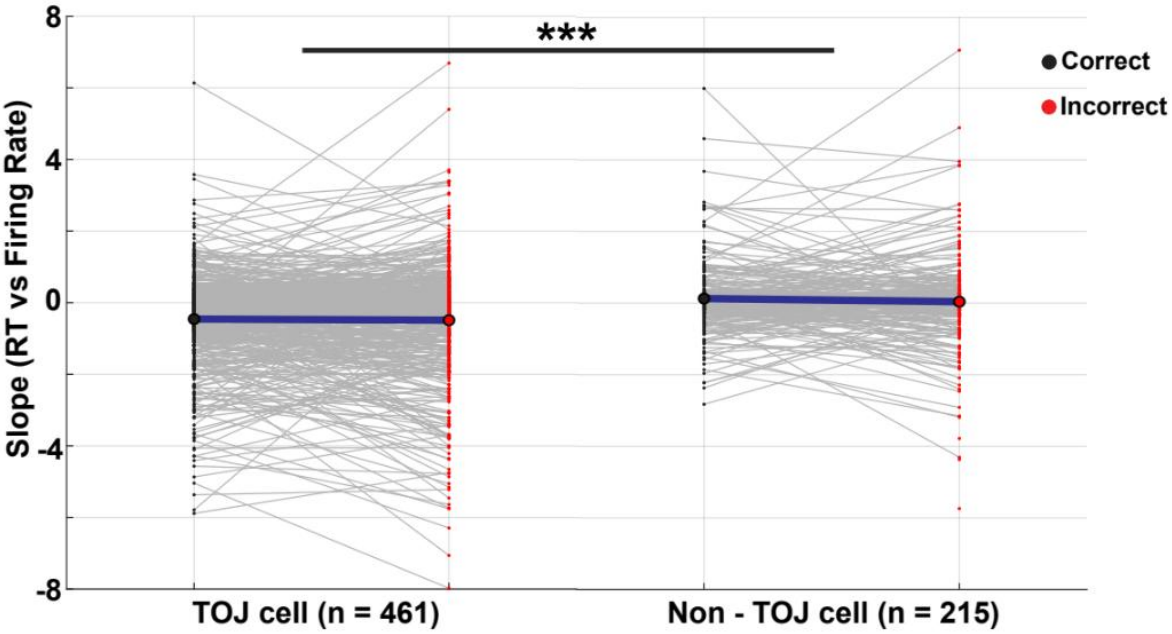

Figure R5. Trial-wise slope of the RT–firing rate relationship per neuron with crossing between neuron category (TOJ vs. non-TOJ) and trial outcome (correct vs. incorrect).

Neither the main effect of trial outcome nor the interaction between neuron category and trial outcome was significant. This suggests that the coupling between TOJ neuron activity and decision speed is present in both correct and incorrect trials, consistent with the interpretation that TOJ neurons reflect the process of temporal order evaluation itself, rather than outcome monitoring or post-decisional signals. The absence of a trial-outcome effect is consistent with human fMRI evidence showing that precuneus activity is engaged during temporal order retrieval independently of accuracy (Kwok et al., 2012). This cross-species convergence supports the interpretation that TOJ neurons reflect temporal order evaluation processes per se, rather than correctness- or feedback-related signals. Importantly, this pattern further argues against explanations based solely on reward, motor execution, or feedback-related activity.

We have incorporated these new analyses and interpretations into the revised Results and Discussion sections (**pp. 5, 11**).

3a). Related to the question above, there are also the following technical issues: 1) do only TOJ neurons show differences between correct and error trials? Is there any difference between correct and error trials for non-TOJ neurons?

Response: We agree that it is important to determine whether firing rate differences between correct and incorrect trials are specific to TOJ neurons. To address this, we performed a linear mixed-effects model analysis in which firing rate during the TOJ period was entered as the dependent variable, with trial outcome (correct vs. incorrect), neuron category (TOJ vs. non-TOJ),

and their interaction as fixed effects, and animal identity as a random effect. This analysis revealed a significant main effect of neuron category ($p < 0.001$), indicating that TOJ neurons exhibit higher firing rates overall than non-TOJ neurons. These results complement the RT–firing rate analysis reported above (Reviewer 3, Major Comment 2) and show that TOJ neurons differ from non-TOJ neurons primarily in their relationship to decision dynamics rather than in outcome-dependent firing rate changes.

For completeness, when including trial outcome as a regressor in the GLM, we identified 72 neurons (~10% of 676 neurons) whose firing rates were significantly modulated by response correctness. Approximately 45% of these neurons belonged to the TOJ category, consistent with the absence of significant interaction between neuron category and trial outcome. These results have been placed in the Supplemental Materials (**Supplemental Text S1**).

3b) The neuronal synchrony results in Figure 5A-B need to be re-examined to rule out the possibility that they are merely byproducts of changes in neuronal firing rates.

Response: We agree that it is essential to rule out the possibility that synchrony effects are driven by firing rate differences. We addressed this concern at multiple levels. First, population firing rates during the TOJ period do not differ between correct and incorrect trials, as demonstrated by a linear mixed-effects model including trial outcome and neuron category (**p. 5**, see Response to Reviewer 3, Major Comment 3a). Second, the synchrony metric used in this study (SPIKE-distance; Kreuz et al., 2015) quantifies relative spike timing independently of firing rate, ensuring that differences in average firing rate do not directly bias synchrony estimates (see also Reviewer 3, Major Comment 3d). In addition, we re-ran the synchrony analyses using permutation-based controls (1,000 permutations; FDR-corrected across time) to verify that the observed SPIKE-distance differences were significantly different from chance (see revised Methods and **updated Figure 3**). Together, these analyses indicate that the observed synchrony effects reflect genuine differences in temporal coordination between neurons rather than trivial firing rate changes (**pp. 6, 21**).

3c) The increase in synchrony at TOJ onset may be driven by the event of stimulus presentation itself, namely an event-induced reset of neuronal activity.

Response: We agree that stimulus onset can induce transient resets of neuronal activity. However, several observations argue against a purely stimulus-driven explanation. First, the synchrony increase differs between experimental conditions (e.g., immediate vs. delayed TOJ), even though the same stimuli are presented, indicating condition-dependent modulation beyond stimulus onset effects. Second, the synchrony dynamics around TOJ offset differ from those at TOJ onset, further suggesting that the observed effects are not simply event-locked resets. We further addressed this possibility by explicitly modeling early visual-onset responses separately from later TOJ-related activity in a control GLM (see Response to Reviewer 2, Major Comment 2). Significant TOJ-related modulation remained after accounting for early sensory transients. Together, these findings argue against a purely stimulus-induced reset and support the interpretation that the observed synchrony increase reflects task-related retrieval and decision processes engaged during temporal order judgment. These clarifications and the additional control analyses have been incorporated into the revised manuscript (**pp. 4-5, 11, 20**).

3d) Similarly, in Figure 5A-B bottom panels, the higher synchrony observed in correct trials raises the question of whether it is simply a consequence of a higher average firing rate in correct trials.

Response: Several lines of evidence argue against this interpretation. First, as described above (Comment 3a), population-level firing rates do not differ between correct and incorrect trials. Second, although a subset of neurons (72/676) shows outcome-related firing rate modulation, these neurons are distributed across 63 recording sessions, making their contribution to session-level synchrony minimal.

Most importantly, the synchrony metric used in our study (SPIKE-distance; Kreuz et al., 2015) quantifies relative spike timing independently of firing rate, ensuring that differences in average firing rate do not directly bias synchrony estimates. Taken together, these results indicate that the higher synchrony observed in correct trials reflects enhanced temporal coordination among neurons rather than trivial firing rate differences. We have clarified this interpretation in the revised manuscript (**pp. 5-6, 11-12, 21**; see also response to Reviewer 1, Major Comment 3).

4. For the results regarding Mahalanobis distances in Fig. 6, comparisons between correct and incorrect trials are welcome, but not sufficient to establish that such distance-based similarities are significantly smaller than chance in the first place. And why not evaluate reactivation directly through one of the myriad methodologies available from rodent (or even human) memory replay/reactivation literature?

Response: We agree with the reviewer that it is essential to establish whether the observed Mahalanobis distance–based similarities differ from chance levels in the first place, beyond comparisons between correct and incorrect trials. To address this, we conducted a permutation analysis to generate a null distribution of distance values.

Specifically, for each session, we independently permuted the firing rate matrices for the encoding and TOJ periods (rows representing trials and columns representing neurons) 1000 times and recomputed the Mahalanobis distance for each permutation. This procedure preserved the marginal firing rate structure while disrupting trial-specific relationships between encoding and retrieval. We found that the Mahalanobis distance indices observed under the experimental conditions were significantly smaller than those from the permuted distributions for both correct and incorrect trials ($P < 0.001$). These results indicate that the similarity between population activity patterns during encoding and retrieval is significantly greater than expected by chance, supporting the involvement of these neuronal populations in temporal-order memory processes.

The results of this permutation analysis are now shown in **updated Figure 6C** and **p. 9** (also Figure R6 below), which demonstrates that Mahalanobis distance indices during the task are significantly smaller than the permuted population in both monkeys and for both trial outcomes.

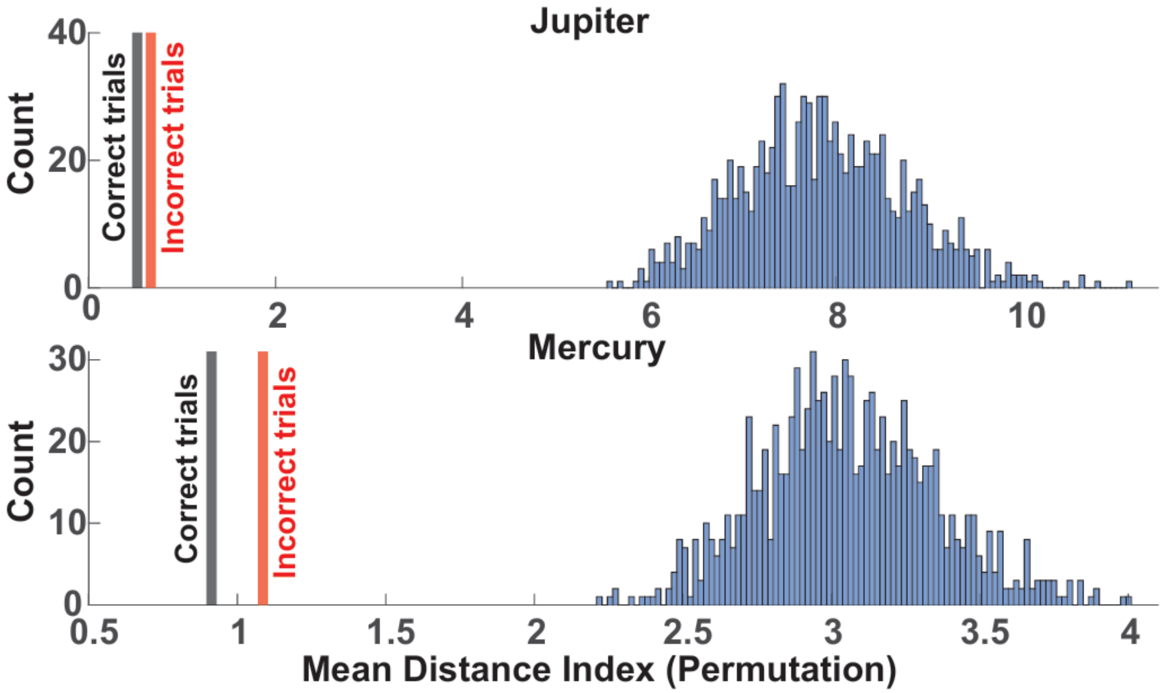

Figure R6. Mahalanobis distance indices between encoding and TOJ periods are significantly smaller than values obtained from permuted data (both correct trials and incorrect trials) in both monkeys.

We thank the reviewer for the suggestion to evaluate reactivation using established replay/reactivation methodologies from the rodent and human memory literature. While we agree that such approaches have been highly informative in many contexts, we did not apply classical replay/reactivation analyses to the present dataset for several reasons. First, the encoding phase in our task involved continuous, naturalistic video stimuli, which lack the discrete, repeated state sequences or trajectories that most replay detection methods critically rely on. Second, canonical replay analyses—such as sequence reactivation during offline rest or sharp-wave ripple–associated events—require extended periods of quiescent or offline activity following learning. In contrast, our study focused on neural dynamics during an active temporal order judgment task, without prolonged rest or sleep epochs during which replay phenomena are typically observed. Although it is conceivable that replay-like analyses could be adapted to examine activity during the brief post-stimulus blank period, such an effort would require substantial methodological development and would raise a distinct set of questions beyond the scope of the present study. We have added a brief discussion of this limitation and its implications in the revised manuscript (**p. 14**).

5. For the following reasons, the results presented in Figure 8 are quite confusing, and it is difficult to understand why the authors conducted these analyses:
The results presented in Figure8 E-H seems to have never been mentioned in the main text. Notably, it appears that the R-squared values in Figure 8E did not show a significant difference between TOJ and non-TOJ neurons. As there is no mention of this and subsequent panels in the Results section, I am uncertain if this is indeed the case. Setting aside the fact that Experiment 2 with two video clips per trial only involved one monkey, the repeated recruitment of TOJ cells at the start of each clip could be interesting if more data were available. However, the proportion of

2-field temporal context neurons seems to be only at a chance level (5%); the authors need to statistically demonstrate that this proportion is significantly higher than chance. Additionally, no further analysis was performed to verify whether these neurons were actually involved in memory encoding. As things stand, the authors' statements, such as " These 2-field temporal context cells are able to more finely encode...", were not well supported.

Response: We thank the reviewer for this detailed and thoughtful critique. We agree that the results presented in Figure 8 were insufficiently integrated into the main Results and that several interpretations—particularly regarding two-field temporal context neurons—were not adequately supported by the available data. Given that Experiment 2 involved only a single animal and carried differences in stimulus structure during the TOJ period (see our response to Reviewer 2, Minor Comment 3) and that the proportion of two-field temporal context neurons did not exceed chance levels, we agree that these analyses should not form part of the main conclusions of the study. To avoid overinterpretation and to maintain a clear focus on the primary findings supported by multi-animal data, we have removed Figure 8 from the main Results and revised the manuscript accordingly. We believe this change improves the clarity and rigor of the manuscript and prevents speculative interpretations that are not yet sufficiently supported.

6. In Figure 1E, why do we see reaction times (RTs) of 90 and 180 frames? Since the monkeys were asked to select the image that appeared earlier, the monkeys should only select frames 5 and 95 in correct trials. So, I suspect that the RTs of 90 and 180 frames were calculated from error trials. If this is the case, then the authors' claim that "RTs were faster for frames chosen from the early part of the video" may not hold. The longer RTs for the later part of the video are likely due to these being error trials.

Response: We thank the reviewer for raising this important point. Reaction time (RT) analyses were intended to assess decision dynamics rather than response accuracy per se. As the reviewer correctly noted, selections of the later frame within each probe pair (e.g., frames 90 or 180) predominantly occur on error trials, which can confound simple comparisons across frame identities. To address this concern directly, we fit a linear mixed-effects model with reaction time as the dependent variable, delay condition (immediate vs. delayed) and trial correctness (correct vs. error) as fixed effects, and monkey identity as a random effect.

RT ~ Delay Condition + Correctness + (1 | Monkey).

This analysis revealed a robust main effect of delay condition, with significantly longer RTs in the delayed condition than in the immediate condition ($p < 0.001$), even after accounting for trial outcome and after controlling for temporal similarity (see Brown et al., 2007). Trial outcome itself also significantly influenced RT ($p < 0.001$), but critically, the effect of delay condition remained robust.

To avoid confusion, we have revised the manuscript to explicitly report this analysis as a comparison between immediate vs. delayed conditions. We have also removed the original description that could be interpreted as conflating frame position with correctness. This clarification is now reflected in the revised Methods and Results sections (**pp. 4, 19**). Please also see our response to Reviewer 3, Major Comment 7, for related clarification.

7. Page 4 states that "…a numeral trend of increased RTs as a function of the four chosen frame locations (Figure 1D)". I could not see how the authors arrived at this conclusion. I suspect the authors might be referring to Figure 1E? However, based on the results, I can only observe that the RT of the latter frame in each pair is greater than that of the former frame. There is no clear evidence of "increased RTs as a function of the four chosen frame locations," especially in the case of monkey Mercury, where no trend as claimed by the authors is apparent.

Response: We thank the reviewer for this careful observation. The reviewer is correct that, with the current four-frame location design, the data does not provide sufficient evidence to support a monotonic increase in reaction times as a function of chosen frame location. We also agree that the original text may have caused confusion regarding the relevant figure reference. A definitive test of whether reaction times scale monotonically with temporal position would require a substantially denser and more finely sampled set of probe frame locations across the video than the limited four-frame design used here (related to Reviewer 3, Major Comment 1). Accordingly, we have removed the statement claiming a "numerical trend of increased RTs as a function of the four chosen frame locations" from the revised manuscript. In the revision, we restrict our interpretation to the more conservative and well-supported comparison between immediate vs. delayed conditions, rather than implying a graded effect across individual frame identities. The relevant text has been revised on **p. 4**. (see also our response to Reviewer 3, Major Comment 6, for related changes).

8. For the decoding method (re: Figures 3A, 3D, and 8F), if I understand correctly, the authors only performed a single test during decoding, where they trained on odd trials and tested on even trials. They did not perform cross-validation by randomly splitting the data into training and testing sets. Typically, cross-validation is necessary, and I would suggest that the authors consider using a standardized cross-validation approach. Common machine learning libraries, such as those provided by "sklearn", offer easy-to-use tools for this purpose to calculate decoding accuracy. Additionally, when generating pseudo-trials, the authors applied a resampling method with replacement to upsample for the sessions with fewer than 200 trials. This could lead to trials with identical values appearing multiple times in the dataset. These identical trials may appear in both the training and testing sets, which could result in an artificially inflated decoding accuracy.

Response: We thank the reviewer for these constructive comments and methodological suggestions. Following them, we implemented two major improvements to our decoding analysis and re-ran all relevant analyses.

First, instead of using a single odd–even split, we now employ a k-fold cross-validation procedure (k = 5) for the LDA decoder. Trials are randomly partitioned into five equally sized folds using a stratified approach to maintain balanced representation across experimental conditions. For each fold, the decoder is trained on four folds (80% of trials) and tested on the remaining fold (20%). This procedure is repeated until each fold has served once as the test set, yielding five independent estimates of decoding performance that are subsequently averaged. Second, to address the reviewer's concern regarding pseudo-trial generation and potential trial duplication across training and testing sets, we eliminated resampling with replacement. Instead, each trial is now used once and only once in the decoding analysis, and the number of trials is matched across neurons by subsampling to the minimum trial count within each monkey (Jupiter: n = 180 trials; Mercury: n

= 90 trials; Mars: n = 120 trials). This ensures that no identical trials appear in both training and testing sets and prevents inflation of decoding accuracy. Specifically, trial indices were randomized using MATLAB's *randperm* function and sequentially assigned to folds. Decoding performance was quantified as mean absolute decoding error and assessed for statistical significance using a permutation test (1000 iterations) with shuffled trial labels to generate a null distribution.

With this revised approach, the LDA decoder successfully classified neural activity into the correct temporal bins, with mean absolute decoding errors of 1.444 s (±0.217 SD across folds) for Jupiter and 1.495 s (±0.251 SD) for Mercury. All decoding performances were significantly above chance ($p < 0.001$). We have updated the Methods and Results sections to reflect these revised analyses and report the new decoding results in the revised manuscript (**updated Figure 5**, **updated Supplemental Figure S2**, and **pp. 8, 22**).

Minor issues:
1. In "Experimental apparatus" subsection under "Experimental model and subject details", "head-refrained" should probably be "head-restrained".

Response: We made corrections to this typo and several other places we noticed as we prepared for this substantially revised manuscript.

2. Figure 1B: tiny white script could be seen above the monkeys' names. Presumably they are not relevant to the manuscript and should be scrubbed. The upper panel of Figure 1B is not labeled with the corresponding monkey's designation.

Response: We thank the reviewer for noting this issue. The extraneous white script above the monkeys' names in Figure 1B has been removed. In addition, the upper panel of **Figure 1B** is now clearly labeled with the corresponding monkey designation. The figure has been updated to ensure consistent and standardized presentation in the revised manuscript.

3. The neurons with long decay are the ones that resemble those in previous work on entorhinal neurons that support temporal context encoding. The authors should include examples of this type of neuron in Figure 2.

Response: We thank the reviewer for this suggestion. We have added one representative example neuron with a long relaxation time to **updated Figure 4A** (middle panel) in the revised manuscript, highlighting neurons that resemble those previously reported to support temporal context encoding. In the **updated Figure 6E**, we also show a number of TOJ ∩ temporal context cells that display long delay profiles during encoding stage.

4. Current supplementary figure S2 spans over two pages; best to resize so it fits on one page.

Response: We thank the reviewer for this suggestion. **Supplemental Figure S2** has been resized to fit on a single page, improving clarity and ease of reading in the revised manuscript.

5. Second-to-last sentence in Results subsection "Spike train synchrony tracks evidence accumulation for TOJ decision" states that "synchrony level is consistently higher for the delayed

condition compared to the immediate condition", while the opposite is true in Fig. 5C-D. The last sentence of the same subsection implies that the authors meant to say that immediate condition yielded greater synchrony, as shown in the figure, but made a mistake in writing.

Response: We thank the reviewer for catching this error. The reviewer is correct that the statement in the second-to-last sentence of this Results subsection was written incorrectly. As shown in **updated Figure 3C–D**, spike train synchrony is consistently higher in the immediate condition than in the delayed condition. The last sentence of the subsection correctly reflects this result. We have now corrected the sentence to accurately describe the data. This was a typographical error in the text only; specifically, we replaced "higher for the delayed condition compared to the immediate condition" with "*Synchrony was higher in the immediate than in the delayed condition when aligned to TOJ onset*" (**p. 6**). We additionally added further clarification in the revised manuscript (see **pp. 11-12**, based on our response to Reviewer 1, Major Comment 3, and Reviewer 2, Minor Comment 2).

6. Some figure labels (e.g., monkey names in Fig. 5) are even larger in font size than the panel labels (A, B, C, etc.).

Response: We appreciate the reviewer's comment. We have standardized the font type and sizes across all panels and figures in the revised manuscript. Specifically, we revised the figures (see also our response to Reviewer 1, Minor Comment 2) and reduced the font size of less critical elements, such as the monkey names, so that panel labels (A, B, C, etc.) are visually emphasized and consistent throughout.

7. The figures and results in Figures 2 and 3 closely resemble those in Ian M. Bright et al.'s work. I would suggest that the authors modify their data presentation and reduce the emphasis on these results, shifting the focus of the paper to the latter sections in order to enhance the novelty of their work. Similarly, the results in Figure 7 are primarily used to rule out additional interference caused by eye movements and do not provide further information supporting the main conclusions. I recommend moving these results to the supplementary results.

Response: We thank the reviewer for this helpful suggestion. In response, we have reorganized the Results section to better highlight the novel aspects of the study and to reduce overlap in emphasis with prior work (e.g., Bright et al.). Specifically, we now place the analyses of temporal context cells and the LDA results after the presentation of the TOJ cell results, thereby shifting the primary focus toward the TOJ-related findings (see also our responses to Reviewer 3, Major Comments 2–5). In addition, because the analyses in the original Figure 7 were intended mainly to rule out potential confounds related to eye movements, we have moved this figure to the Supplementary Materials (**Supplemental Figure S3**).

8. Regarding Poisson GLM models: why not show the full thing instead of just the R2 distributions as in Fig. 4A/Fig. 8E? Is TOJ actually the most important variable? The clear difference among TOJ cell distribution shapes across monkeys also does not inspire confidence; are those cells truly "TOJ cells" even with $R2 < 0.1$ as in many neurons of monkey Jupiter? If stepwise GLM was performed according to the Methods, perhaps the formulae for the final models could be shown to assuage such doubts?

Response: We thank the reviewer for raising these important points regarding Poisson GLM analysis and the interpretation of TOJ cells. We agree that additional clarification of the modeling procedure and variable contributions would improve transparency.

With respect to whether TOJ is the most important variable in the model, we emphasize that our goal was not to rank predictors by variance explained, but to identify neurons whose activity was significantly modulated during the TOJ epoch after accounting for other task events. In line with this goal, we used a stepwise GLM procedure, as described in the Methods and consistent with the literature (Tsao et al., 2018), to determine the final model for each neuron. We included the full GLM now in the revised manuscript (see Figure R7 below and **updated Figure 2A**). We have clarified this explicitly in the Methods section and provided the corresponding model formulae in the revised manuscript (**p. 20**).

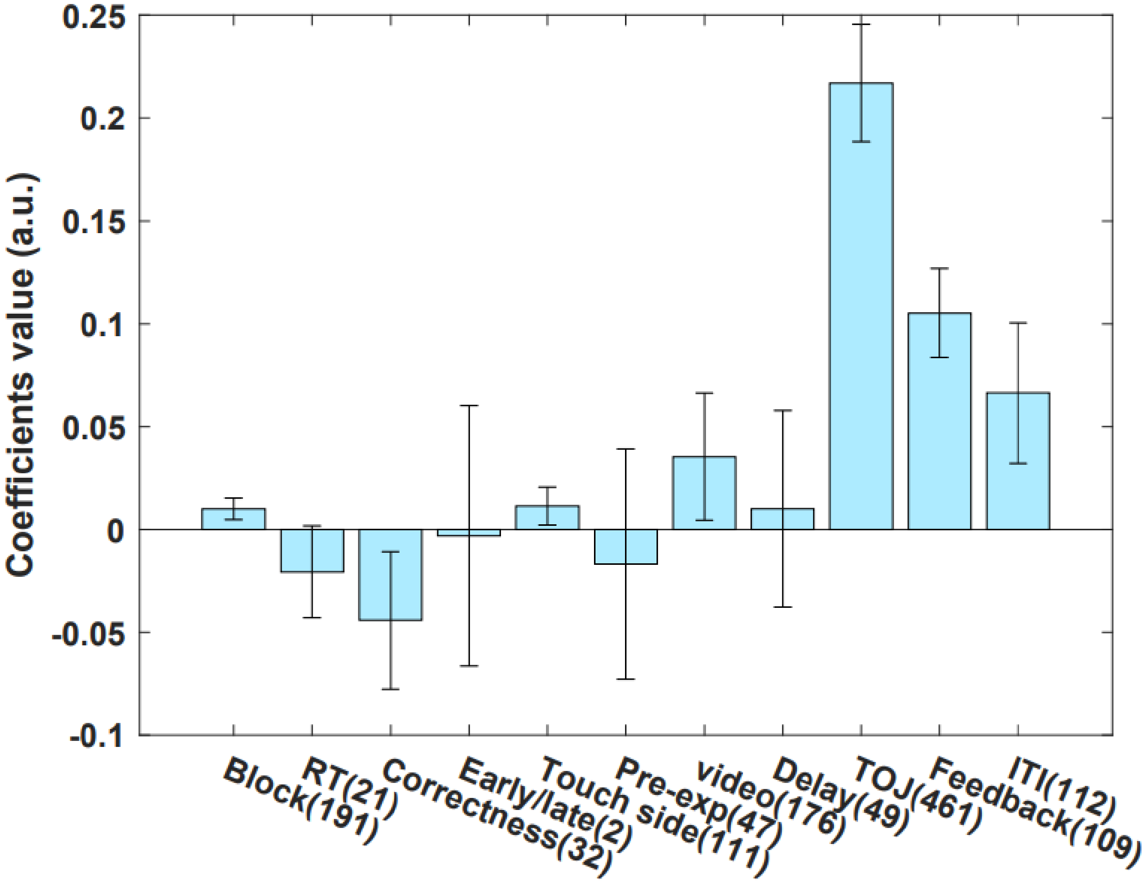

Figure R7. GLM result for various task variables. Numerals in bracket indicate the number of neurons that are statistically modulated by specific variables.

Regarding presentation of the GLM results, our original intention in showing the distribution of pseudo-R^2 values (Figure 4A in original submission) was to provide a summary measure of overall model fit rather than to imply that TOJ-related modulation is driven by large effect sizes at the level of individual neurons. We agree that pseudo-R^2 values are generally small for Poisson GLMs applied to single-neuron spike data, particularly in naturalistic tasks, and low R^2 values do not

preclude statistically reliable modulation by specific task variables. Importantly, neurons were not classified as TOJ cells based on R^2 magnitude, but rather on the statistical significance of the TOJ-related regressors within the GLM. Thus, neurons with relatively small overall pseudo-R^2 values can still exhibit reliable TOJ-related modulation. Differences in the shape of the R^2 distributions across monkeys likely reflect inter-animal variability in firing statistics and recording conditions, rather than differences in the validity of TOJ cell classification. We have revised the Results text to clarify that TOJ cell classification reflects statistically reliable TOJ-related modulation rather than large effect sizes, and we have tempered language that could be interpreted as implying otherwise. We believe these changes address the reviewer's concerns and improve the clarity and interpretability of the GLM analyses (**pp. 4–5**).

References:

Brown, G. D. A., Neath, I., & Chater, N. (2007). A temporal ratio model of memory. Psychological Review, 114(3), 539–576. https://doi.org/10.1037/0033-295X.114.3.539

Kreuz T, Mulansky M, Bozanic N. (2015). SPIKY: A graphical user interface for monitoring spike train synchrony. J Neurophysiol. 113(9):3432-3445.

Kwok SC, Shallice T, Macaluso E. (2012). Functional anatomy of temporal organisation and domain-specificity of episodic memory retrieval. Neuropsychologia. 50(12):2943-2955. doi:10.1016/j.neuropsychologia.2012.07.025

Tsao A, Sugar J, Lu L, et al. Integrating time from experience in the lateral entorhinal cortex. Nature. 2018;561(7721):57-62. doi:10.1038/s41586-018-0459-6
