# Supplementary material for: Neural population dynamics and temporal context cells in macaque medial parietal cortex support temporal order memory
Source: PLoS Biol. 2026 Apr 17;24(4):e3003759. doi: 10.1371/journal.pbio.3003759 (PMC13108878; doi:10.1371/journal.pbio.3003759)
Supplement: Attachment — Submitted filename: Responses to Reviewer_R3.docx [file pbio.3003759.s011.docx]

Reviewer #1: The authors have sufficiently addressed all my concerns, and I recommend the paper for publication.

Response: We sincerely thank the reviewer for the positive evaluation and for the helpful and constructive feedback provided throughout the review process, which has greatly improved the manuscript.

Reviewer #3 (Liping Wang has signed his report): In this revision, the authors went to great lengths to address my concerns, and adequately so for the most part. While I appreciate the substantial effort evident from the overhaul of the manuscript, I do still have a few lingering issues that would hopefully improve the article further if properly addressed.

1. Regarding major issue #6 in our original review, since the authors have revised the results in Figure 1E to focus on comparing reaction time (RT) differences between the immediate and delayed conditions, I suggest they accordingly update the presentation of Figure 1E. Specifically, they should clearly label the groups being compared (The key comparison should be Frame 5 vs. Frame 90, right?) and include the corresponding statistical results to facilitate reader understanding. Furthermore, since the 90- and 180-frame trials represent error trials and the primary objective appears to be comparing immediate vs. delayed conditions, it seems unclear why these error trials were included in this analysis. Notably, while the figure displays results for the two monkeys separately, the main text appears to report statistical results based on pooled data from both animals. Does the conclusion stated in the main text also hold true for monkey Mercury? Generally speaking, I recommend that the authors double-check their revisions to ensure consistency between the figure(s) and the main text.

Response: We thank the reviewer for this helpful suggestion and have revised Figure 1E and the corresponding text to improve clarity and consistency. We have now explicitly labeled the compared frame positions (immediate vs delay, e.g., Frame 5 vs Frame 95) in the figure to clarify the key comparison. We agree that inclusion of error trials may have been confusing. We have clarified in the revised figure legend and text that the analysis focuses on correct trials for the main comparison, while error trials are shown for completeness. We have ensured consistency between the figure and the main text by explicitly indicating when results are shown separately for each monkey and when statistical analyses are performed on pooled data.

2. While the video time decoding and encoding-retrieval similarity analyses yielded encouraging results, they appear only tangentially related to previous classification efforts that identified populations of TOJ/temporal context cells. One is left wondering, for instance, whether temporal context cells seen in Figure 4 preferentially contributed to decoding in Figure 5, or how distance metrics in Figure 6 would be impacted when considering only temporal information-carrier neurons versus others. This disjunction, alongside newly introduced sidetracking sections that addressed various issues brought up by reviewers, made reading through the manuscript a fairly fragmentary experience. Perhaps the authors should consider shoring up their main narrative throughout the Results section, so that the readers could follow along a logical chain of events that would bridge the gap between cell type identification and task information representation.

Response: We thank the reviewer for this important suggestion and agree that the connection between cell-type identification and population-level analyses could be clarified. In the revised manuscript, we have strengthened the narrative throughout the Results section by adding explicit bridging text at key transition points. Specifically, we now (i) clarify at the end of the temporal context cell analysis how these single-neuron properties relate to subsequent population-level decoding analyses, (ii) explicitly state at the beginning of the decoding section that these analyses reflect distributed population activity rather than being restricted to temporal context cells, and (iii) link the encoding–retrieval similarity analysis to these population-level representations. In addition, we clarify that the overlap between temporal context cells and TOJ cells is consistent with chance-level independence (Table S1), emphasizing that these functional properties are not preferentially co-localized at the single-neuron level. Together, these revisions provide a clearer and more continuous account linking cell-type identification, population-level dynamics, and temporal order evaluation.

3. Figure 2A, the text of the ticks on the x-axis is overlapping with the axis line. The bottom edge of Figure 5G was cropped off.

Response: We thank the reviewer for pointing out these presentation issues. We have corrected the formatting issues noted by the reviewer, including the overlapping x-axis labels in Figure 2A and the cropped panel in Figure 5G.
