# Supplementary material for: Neural population dynamics and temporal context cells in macaque medial parietal cortex support temporal order memory
Source: PLoS Biol. 2026 Apr 17;24(4):e3003759. doi: 10.1371/journal.pbio.3003759 (PMC13108878; doi:10.1371/journal.pbio.3003759)
Supplement: Attachment — Submitted filename: ResponseLetter_R4.docx [file pbio.3003759.s012.docx]

Based on our Academic Editor's assessment of your revision, we are likely to accept this manuscript for publication, provided you satisfactorily address the following data and other policy-related requests:

* We would like to suggest a different title to improve its accessibility for our broad audience:
Temporal context cells in the macaque medial parietal cortex integrate temporally extended experience with memory-guided decisions

Response: We thank the editor for this helpful suggestion. We agree with the goal of improving accessibility for a broad audience. At the same time, we aimed to ensure that the title accurately reflects the central contribution of the study, which focuses on temporal order memory and retrieval processes, as emphasized during revision.

With this in mind and following an email exchange with Dr Christian Schnell (Senior Editor), we have adopted the following revised title:

**“Neural population dynamics and temporal context cells in macaque medial parietal cortex support temporal order memory.”**

We believe this title maintains accessibility while more directly reflecting the main findings of the manuscript.

* Please add the links to the funding agencies in the Financial Disclosure statement in the manuscript details.

Response: We have updated the Financial Disclosure statement to include the relevant funding agencies along with their corresponding links, in accordance with journal requirements.

* DATA POLICY:

You may be aware of the PLOS Data Policy, which requires that all data be made available without restriction: <http://journals.plos.org/plosbiology/s/data-availability>. For more information, please also see this editorial: <http://dx.doi.org/10.1371/journal.pbio.1001797>

Note that we do not require all raw data. Rather, we ask that all individual quantitative observations that underlie the data summarized in the figures and results of your paper be made available in one of the following forms:

1) Supplementary files (e.g., excel). Please ensure that all data files are uploaded as 'Supporting Information' and are invariably referred to (in the manuscript, figure legends, and the Description field when uploading your files) using the following format verbatim: S1 Data, S2 Data, etc. Multiple panels of a single or even several figures can be included as multiple sheets in one excel file that is saved using exactly the following convention: S1_Data.xlsx (using an underscore).

2) Deposition in a publicly available repository. Please also provide the accession code or a reviewer link so that we may view your data before publication.

Response: We thank the editor for these instructions and have fully complied with the PLOS Data Policy.

All individual numerical data underlying the results presented in the manuscript have been made available as Supporting Information in the file:

**S1_Data.xlsx**

Regardless of the method selected, please ensure that you provide the individual numerical values that underlie the summary data displayed in the following figure panels as they are essential for readers to assess your analysis and to reproduce it: 1E, 2ABDEF and 6B.

NOTE: the numerical data provided should include all replicates AND the way in which the plotted mean and errors were derived (it should not present only the mean/average values).

Response: The file **S1_Data.xlsx** contains the individual-level data underlying all specified figure panels (Figures 1E, 2A, 2B, 2D, 2E–F, and 6B), including:

All individual observations (trial-level or neuron-level data, as appropriate)

Relevant grouping variables (e.g., monkey, session, condition, neuron category, trial outcome)

The values used to compute all reported summary statistics

The dataset enables full reproduction of the analyses and figures.

Please also ensure that figure legends in your manuscript include information on where the underlying data can be found, and ensure your supplemental data file/s has a legend.

Please ensure that your Data Statement in the submission system accurately describes where your data can be found.

Response: We have updated the relevant figure legends to include the statement:

“Data underlying this figure is available in S1 Data.”

We have also updated the Data Availability Statement in the submission system to accurately describe the location and contents of the data.

* CODE POLICY

Per journal policy, if you have generated any custom code during the course of this investigation, please make it available without restrictions. Please ensure that the code is sufficiently well documented and reusable, and that your Data Statement in the Editorial Manager submission system accurately describes where your code can be found. More information on our Code Policy, what and how to share can be found here: <https://journals.plos.org/plosbiology/s/code-availability>

Please note that we cannot accept sole deposition of code in GitHub, as this could be changed after publication. However, you can archive this version of your publicly available GitHub code to Zenodo. Once you do this, it will generate a DOI number, which you will need to provide in the Data Accessibility Statement (you are welcome to also provide the GitHub access information). See the process for doing this here: <https://docs.github.com/en/repositories/archiving-a-github-repository/referencing-and-citing-content>

Response: Custom code used for data analysis has been made publicly available. The code has been archived on Zenodo to ensure a permanent and citable record. The archived version corresponds to the code used in this study and is fully documented to enable reuse. A link to the associated repository is provided in the Data Availability Statement.

**Data and code availability.** Analysis code and processed data supporting the conclusions of this study are deposited on Zenodo, see <https://doi.org/10.5281/zenodo.19280257>

* We do not have a word count limit. Please move the supplementary text to the main manuscript, so readers can easily access this information.

Response: We have merged the supplementary text (Supplemental Text S1) into the main manuscript to improve accessibility and ensure that all relevant analyses are presented in the primary text.

As you address these items, please take this last chance to review your reference list to ensure that it is complete and correct. If you have cited papers that have been retracted, please include the rationale for doing so in the manuscript text, or remove these references and replace them with relevant current references. Any changes to the reference list should be mentioned in the cover letter that accompanies your revised manuscript.

Response: We have carefully reviewed the reference list to ensure that all citations are complete, accurate, and appropriately matched to the content of the manuscript. No retracted references are included. No substantive changes to the reference list were required.
